# Selecting optimal spectral bands for improved detection of autofluorescent biomarkers in multiphoton microscopy

**DOI:** 10.1117/1.JBO.25.7.071206

**Published:** 2020-07-07

**Authors:** Björn-Ole Meyer, M. Pilar J. Stella, Bjørn Holst, Boye S. Nielsen, Kim Holmstrøm, Peter E. Andersen, Dominik Marti

**Affiliations:** a Technical University of Denmark, DTU Health Tech, Roskilde, Denmark; b Bioneer A/S, Hørsholm, Denmark

**Keywords:** multiphoton microscopy, two-photon excitation fluorescence, spectroscopy, fluorescence spectrum, biomarkers, hyperspectral imaging, functional imaging, redox ratio

## Abstract

**Significance**: In multiphoton microscopy, two-photon excited fluorescence (TPEF) spectra carry valuable information on morphological and functional biological features. For measuring these biomarkers, separation of different parts of the fluorescence spectrum into channels is typically achieved by the use of optical band pass filters. However, spectra from different biomarkers can be unknown or overlapping, creating a crosstalk in between the channels. Previously, establishing these channels relied on prior knowledge or heuristic testing.

**Aim**: The presented method aims to provide spectral bands with optimal separation between groups of specimens expressing different biomarkers.

**Approach**: We have developed a system capable of resolving TPEF with high spectral resolution for the characterization of biomarkers. In addition, an algorithm is created to simulate and optimize optical band pass filters for fluorescence detection channels. To demonstrate the potential improvements in cell and tissue classification using these optimized channels, we recorded spectrally resolved images of cancerous (HT29) and normal epithelial colon cells (FHC), cultivated in 2D layers and in 3D to form spheroids. To provide an example of an application, we relate the results with the widely used redox ratio.

**Results**: We show that in the case of two detection channels, our system and algorithm enable the selection of optimized band pass filters without the need of knowing involved fluorophores. An improvement of 31,5% in separating different 2D cell cultures is achieved, compared to using established spectral bands that assume NAD(P)H and FAD as main contributors of autofluorescence. The compromise is a reduced SNR in the images.

**Conclusions**: We show that the presented method has the ability to improve imaging contrast and can be used to tailor a given label-free optical imaging system using optical band pass filters targeting a specific biomarker or application.

## 1 Introduction

Two-photon excitation fluorescence microscopy (TPEFM) is an established tool in the biosciences^1^ and a promising modality for clinical diagnostics.^2^ It is used in the neurosciences to investigate calcium dynamics, neuronal plasticity, and neurodegenerative diseases, and in cancer research for *in vivo* studies, as well as in immunology and embryology.^1^ It is capable of probing endogenous biomarkers^3^ with no or limited photodamage,^4^ thus enabling label-free optical imaging potentially suitable as a minimally invasive cancer diagnostic tool in an endoscope.^2^ The obtained morphological information compares well to pathological examinations on hematoxylin–eosin-stained biopsy slides^5^ and can yield additional information, e.g., intracellular features such as the nuclear density ratio.^6^ Furthermore, TPEFM reveals functional information inaccessible by current methods, such as cellular secretion, relevant in the neurosciences,^7^ or *in vivo* mapping of metabolic changes,^8^ as well as observing of drug-induced or endogenous porphyrin fluorescence for early-stage cancer diagnostics and photodynamic treatment.^9^^,^^10^

Among the endogenous fluorophores available in TPEFM, reduced nicotinamide adenine dinucleotides [NAD(P)H] and oxidized flavin adenine dinucleotides (FAD) are considered major contributors to autofluorescence^2^ and are often used to create a redox ratio.^8^^,^^11^^–^^15^ NAD(P)H and FAD play an important role in glycolysis, the Krebs cycle, and oxidative phosphorylation; therefore, ratiometric measurements of their absolute or relative concentrations provide information about cell or tissue metabolism,^14^ which correlates well with the established Seahorse flux analysis.^16^

To interpret the two-photon excited fluorescence (TPEF) signals, the emitted fluorescence light is typically separated into multiple spectral channels by selecting appropriate optical filters. The selection of optical filters requires knowledge of the spectral composition of the acquired signals. NAD(P)H and FAD, for example, can be isolated by sequential excitation of the sample at 750 and 900 nm, or by simultaneous acquisition with 410- to 480-nm and 510- to 560-nm bandpass filters using 800-nm excitation.^17^ The latter approach would be more appropriate for use of TPEFM as a diagnostic method in a clinical workflow, where cost effectiveness and speed are key.^18^ Simultaneous biomarker detection with one excitation wavelength has also been suggested in fluorescence lifetime imaging to improve temporal resolution and motion artifacts.^19^ The filter set in a diagnostic system has to allow the best possible diagnostic accuracy. While the evaluation of those and similar bands lead to above-mentioned findings, the establishment of those bands relies on measurements of NAD(P)H and FAD in solution.^3^^,^^17^ In a complex intracellular environment, endogenous fluorescence depends on a variety of influences, out of which the ratio of bound to free NAD(P)H has been identified as the main contributor.^20^ As a result, fluorescent emissions may change in intensity and spectral shape.^14^ Linear unmixing of TPEFM spectroscopy acquisitions on mesenchymal stem cells showed a blueshifted NAD(P)H and redshifted FAD emission, but nevertheless verified the ability to differentiate NAD(P)H, lipofuscin, and FAD using two excitation wavelengths and two collection channels.^15^ Additionally, the extracellular influences, such as the pH value, may have an effect on the emission spectra, as this is known from measurements on porphyrins in different solutions.^21^ As such, there is a need for a method to define optimal filter sets that allow to accurately classify cells and tissue into “normal” and “abnormal” for a given disease if one wishes to apply TPEFM as a diagnostic method in the clinic. Up to now, no quantitative investigation of separation and signal collection efficiency for desired biomarkers has been published to our knowledge.

In this paper, we substantiate the above findings and establish a method to quantify the separation and the signal collection efficiency of spectral bands for any given disease. To this avail, we have built a spectrally resolved multiphoton microscope to record hyperspectral TPEFM images and designed an algorithm that suggests optimal spectral bands for a given application. We demonstrate the capabilities of this method using two-dimensional (2D) and three-dimensional (3D) cell cultures of cancerous and normal cell lines of colon epithelial cells and confirm that the microenvironment of the cells has an influence on the fluorescence spectra and thus the choice of appropriate spectral bands. Ultimately, this method enables an informed choice of filters for diagnostic and research purposes.

## 2 Materials and Methods

### 2.1 Hyperspectral Multiphoton Microscopy System

**Fig. 1:**
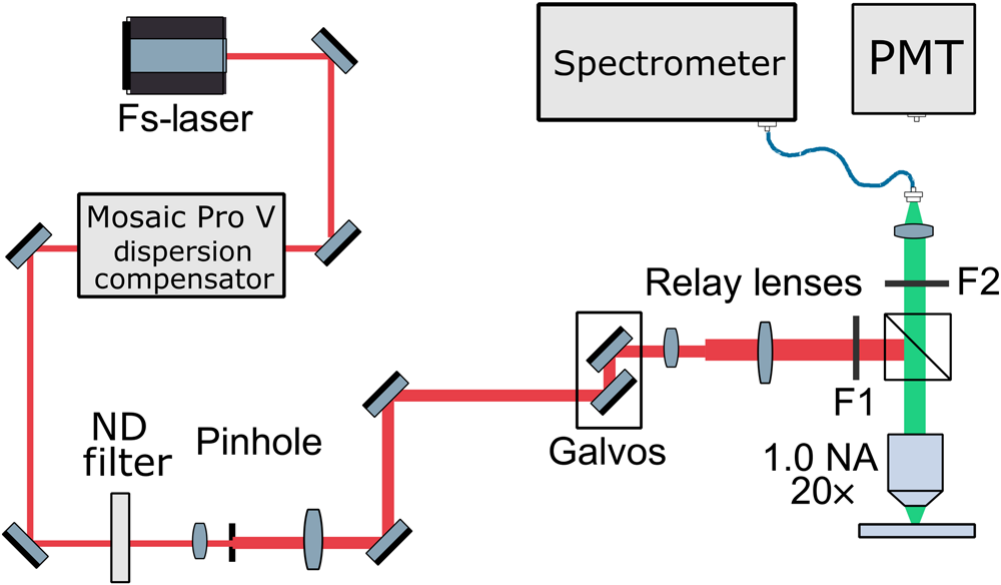
Setup of the custom-built two-photon fluorescence microscope. As excitation source, we use a mode-locked Ti:sapphire laser with a center wavelength at 785 nm for illumination. The emitted fluorescence light is collected in epi-detection by the same objective and then focused into a fiber. The signals can be detected by a PMT for quick imaging or a spectrometer for hyperspectral imaging.

The system is a custom-built multiphoton microscope, as shown in Fig. 1 and discussed in the Supplemental Material in greater detail. Briefly, a femtosecond laser with a center frequency at 785 nm and a pulse length of 15 fs is used for illumination. After passing a dispersion precompensation unit, the beam is sent through a spatial filter before being redirected by a galvanometer scanner and two relay lenses. The beam passes a longpass ﬁlter, is reflected by a dichroic mirror, and is focused with a water dipping objective. Emitted light is collected through the same objective, filtered by the dichroic mirror and a 720-nm shortpass ﬁlter, before being focused into a fiber with a 600-μm core. The signals can then either be detected by a photomultiplier tube (PMT) for quick imaging or a spectrometer (QE Pro, Ocean Insight, Orlando, FL) for hyperspectral imaging. The setup is controlled by a custom LabVIEW program. For hyperspectral imaging, an average power of 12 mW on the samples was used with a pixel dwell time of 8 ms, whereas imaging with the PMT was conducted at 2.3 mW with 100-ns pixel dwell time to avoid photobleaching. The spectral shape was not significantly influenced by photobleaching, as shown in Fig. S5(a) in the Supplementary Material.

### 2.2 TPEF Spectra Processing

High-resolution images of the samples are recorded for navigation using the PMT. Hyperspectral TPEF images are then recorded with reduced resolution ($64\times 64\;{\mu m}^{2}$, $64\times 64\;{\mathrm{pixels}}^{2}$). The spectra of the entire TPEF images are averaged over all pixels. Given by the selected spectrometer slit, the spectral resolution of the system is 10 nm, while the spectrometer has a pixel size covering <0.5  nm per pixel. Therefore, the recorded spectra were smoothed with a sliding window of 10 nm width. All spectra are background subtracted and compensated for a temperature offset using Spectragryph,^22^ as described in Fig. S1 in the Supplementary Material. The investigated spectral range is limited from 425 to 650 nm in which a linear system response function has been confirmed and laser excitation is excluded, as shown in Figs. S2 and S3 in the Supplementary Material, respectively.

Three samples of each cancer model and cell line (detailed below) are investigated, and their mean spectra are calculated and used as an input for the algorithm in the presented analysis. Additionally, spectra from the literature are investigated. The TPEF spectra of NAD(P)H and FAD as reported by Huang et al.^17^ are extracted using WebPlotDigitizer.^23^

### 2.3 Algorithm for Optimal Filter Determination

The presented algorithm takes two spectra as input and quantifies, for all possible spectral band combinations, the ability to separate and thus classify the two spectra, corresponding to the achievable contrast of an imaging system, and the resulting signal-to-noise ratio (SNR) normalized to the maximum possible SNR of the system. It is expected that a trade-off between these two parameters will have to be made, as for many known endogenous fluorophores, fluorescence emission spectra are overlapping.

**Fig. 2:**
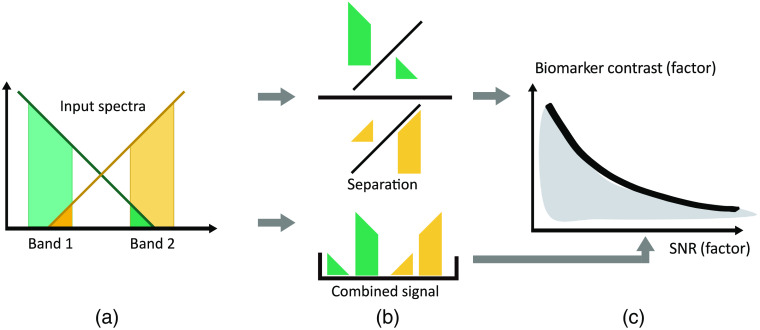
Infographic visualizing the underlying algorithm. (a) Two input spectra (orange and dark green) are separated into spectral bands. (b) The ratios of the power falling into these spectral bands (yellow and light green) are calculated separately for each spectrum, and the two ratios are then divided to quantify the separation. (c) The separation is compared against the geometric mean of the four band’s powers, which serves as a measure for the expected SNR of the entire system.

Figure 2 shows an overview of the algorithm. To calculate the separation of a given filter setup, ideal bandpass filters are assumed, and the probability mass function (PMF) of each spectrum is summed in the spectral bands, from $\lambda_{\mathrm{SB}1,\mathrm{start}}$ to $\lambda_{\mathrm{SB}1,\mathrm{stop}}$, as shown for band 1 in spectrum 1 in the following equation:

$${\mathrm{band}}_{1,S1}=\overset{\lambda_{\mathrm{band}1:\mathrm{end}}}{\underset{\lambda_{\mathrm{band}1:\mathrm{start}}}{\sum }}\mathrm{spectrum}1.$$ (1)

Subsequently, the ratio of the two spectral bands is created within each spectrum to represent the relative change within the signal independent from its absolute intensity. This reduces the influences of variations in the system performance.

$${\mathrm{ratio}}_{S1}=\frac{{\mathrm{band}}_{1,S1}}{{\mathrm{band}}_{2,S1}}.$$ (2)

The ratios of the spectral bands in the two spectra are then divided to calculate the separation.

$$\frac{{\mathrm{ratio}}_{S1}}{{\mathrm{ratio}}_{S2}}\geq 1\to \mathrm{Separation}=\frac{{\mathrm{ratio}}_{S1}}{{\mathrm{ratio}}_{S2}},\;\frac{{\mathrm{ratio}}_{S1}}{{\mathrm{ratio}}_{S2}}<1\to \mathrm{Separation}=\frac{{\mathrm{ratio}}_{S2}}{{\mathrm{ratio}}_{S1}}.$$ (3)

The inverse is taken if the ratio of ratios is smaller than 1, as in this case, the bands only need to be “swapped” to provide a high separation.

To quantify the relative signal collection efficiency using a given filter setup, the geometric mean of the power in the four spectral bands is calculated. Using the geometric mean of the four spectral bands (two in each spectrum), the algorithm prefers spectral bands with balanced signal strengths in both spectra, avoiding small (in bandwidth) or weak (in signal) bands that would have very low SNRs. The resulting relative signal collection efficiency will range from 0 to 0.5, as in the most balanced case each spectral band would contain half of the total signal in each spectrum. To predict the SNR achievable with the determined spectral bands, the SNR of the entire recorded spectrum can be scaled by this factor.

$$\text{relative signal collection efficiency}=\sqrt[{4}]{{\mathrm{band}}_{1,S1}*{\mathrm{band}}_{2,S1}*{\mathrm{band}}_{1,S2}*{\mathrm{band}}_{2,S2}.}$$ (4)

Both the separation and the relative signal collection efficiency are calculated for all permutations of the four wavelengths $\lambda_{\mathrm{band}1:\mathrm{start}},\lambda_{\mathrm{band}1:\mathrm{end}},\lambda_{\mathrm{band}2:\mathrm{start}},\mathrm{and}\;\lambda_{\mathrm{band}2:\mathrm{end}}$ for which the two spectral bands do not overlap, i.e., where $\lambda_{\mathrm{band}1:\mathrm{end}}<\lambda_{\mathrm{band}\;2:\;\mathrm{start}}$. The resulting values for all possible spectral bands can then be visualized in a scatter plot, with the relative signal collection efficiency on the x axis and the separation on the y axis (see Fig. 5 and subsequent figures).

TPEF emission spectra (and, in fact, any type of spectra) can be imported and all possible filter setups within a given detection range are calculated. Custom filter setups, such as filters suggested in literature or readily available filters, can be highlighted in the graph. By selecting spots on the graph, their filter combinations are revealed.

The MATLAB code is available at https://gitlab.gbar.dtu.dk/biophotonics/optimizeSpectralChannels/.

### 2.4 Cell Models

Cancerous and normal epithelial colon cells, namely human epithelial colon adenocarcinoma cells (HT29) and human fetal epithelial colon cells (FHC), were cultivated at Bioneer A/S, Denmark, as 2D cultures and spheroids. Spheroids are known to mimic an *in vivo* cell behavior concerning spatial configuration and signaling pathways.^24^ For the formation of spheroids, 25,000 cells (subcultured for 7 days before trypsinization) and 100  μl of cell medium were used per well in an ultralow attachment round bottom 96-well plate (Corning), which was spun at 500 rpm for 10 min. The 2D cultures were seeded with 25,000 cells and 2  μl per petri dish. The cell medium used was Dulbecco’s Modified Eagle’s Medium with 4.5 g l-glucose per liter, 10% fetal bovine serum (20% FBS for FHC cells), 1% of penicillin/streptomycin solution and 1% of L-glutamine. 2D cultures and spheroids for biomarker detection were kept in a normal oxygen incubator (37°C, 5% ${\mathrm{CO}}_{2}$, 20% $O_{2}$) for 9 days and then transferred to a low oxygen incubator (37°C, 5% ${\mathrm{CO}}_{2}$, 5% $O_{2}$) to create a cancer characteristic tissue environment and harvested after another 3 days to avoid excess buildup of metabolic waste. The development of spheroids was monitored using optical coherence tomography (OCT) to optimize the sample consistency and time of harvesting.

**Fig. 3:**
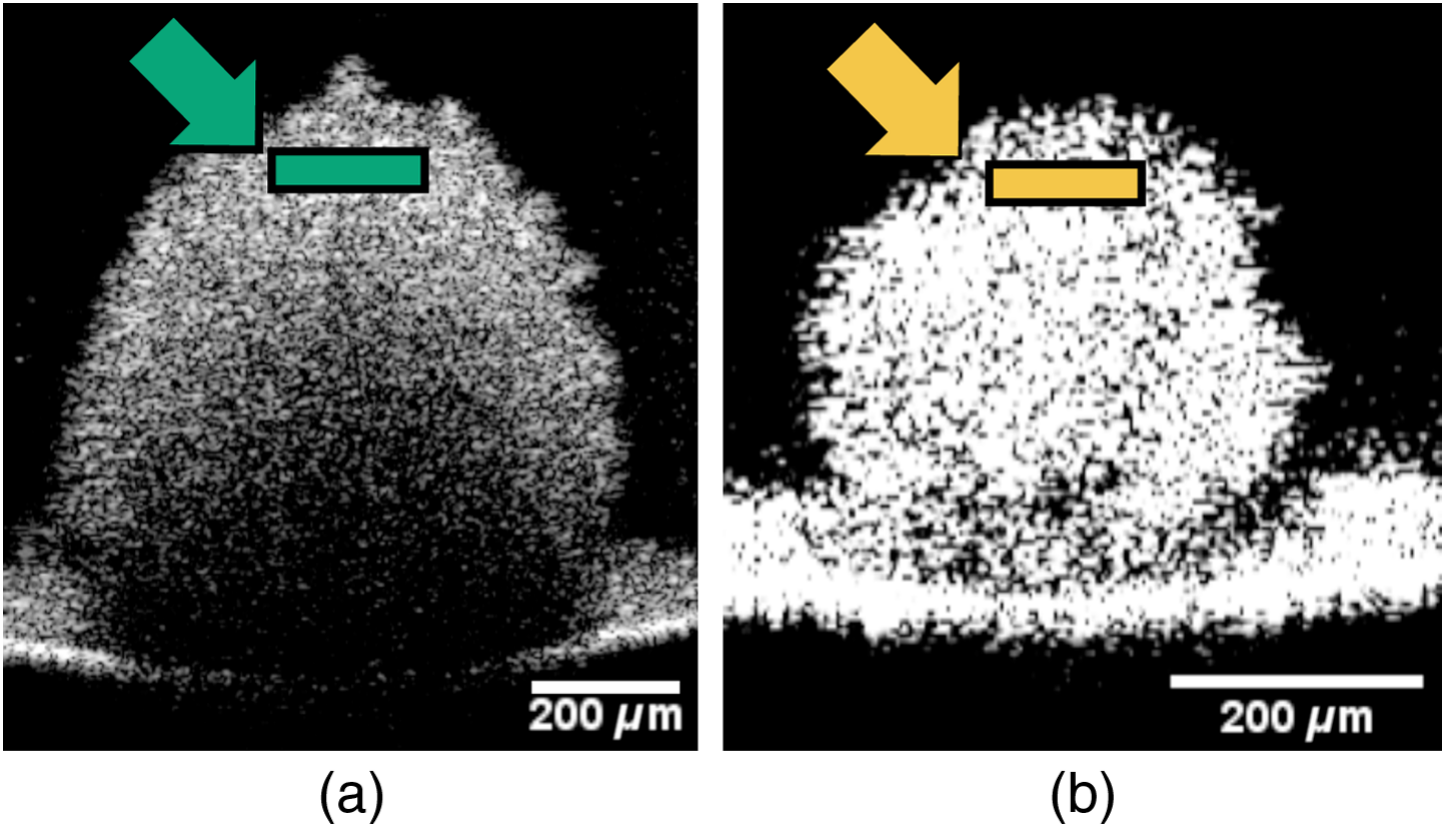
(a) Cancerous (HT29) and (b) normal (FHC) epithelial colon cells are grown as 3D spheroids. Spheroid formation is observed using a commercial OCT system (TELESTO-II, THORLABS), and approximate TPEFM imaging locations are marked (arrow).

For the imaging of 2D cultures, three locations for TPEFM acquisition were chosen randomly in one petri dish for each cell type. For spheroids, three samples of each cell type were imaged, and locations were chosen centrally, about two cell layers deep to yield a homogenous layer of cells while avoiding debris on the surface and necrotic regions deeper inside the spheroid. Approximate imaging areas are shown in Fig. 3. A further set of 2D cultures and a 2D co-culture using both cell lines have been grown for 7 days in a normal oxygen and 4 days in a low-oxygen incubator to demonstrate the application.

## 3 Results

### 3.1 Two-Photon Excited Fluorescence Emission Spectra of Live Cell Cultures

**Fig. 4:**
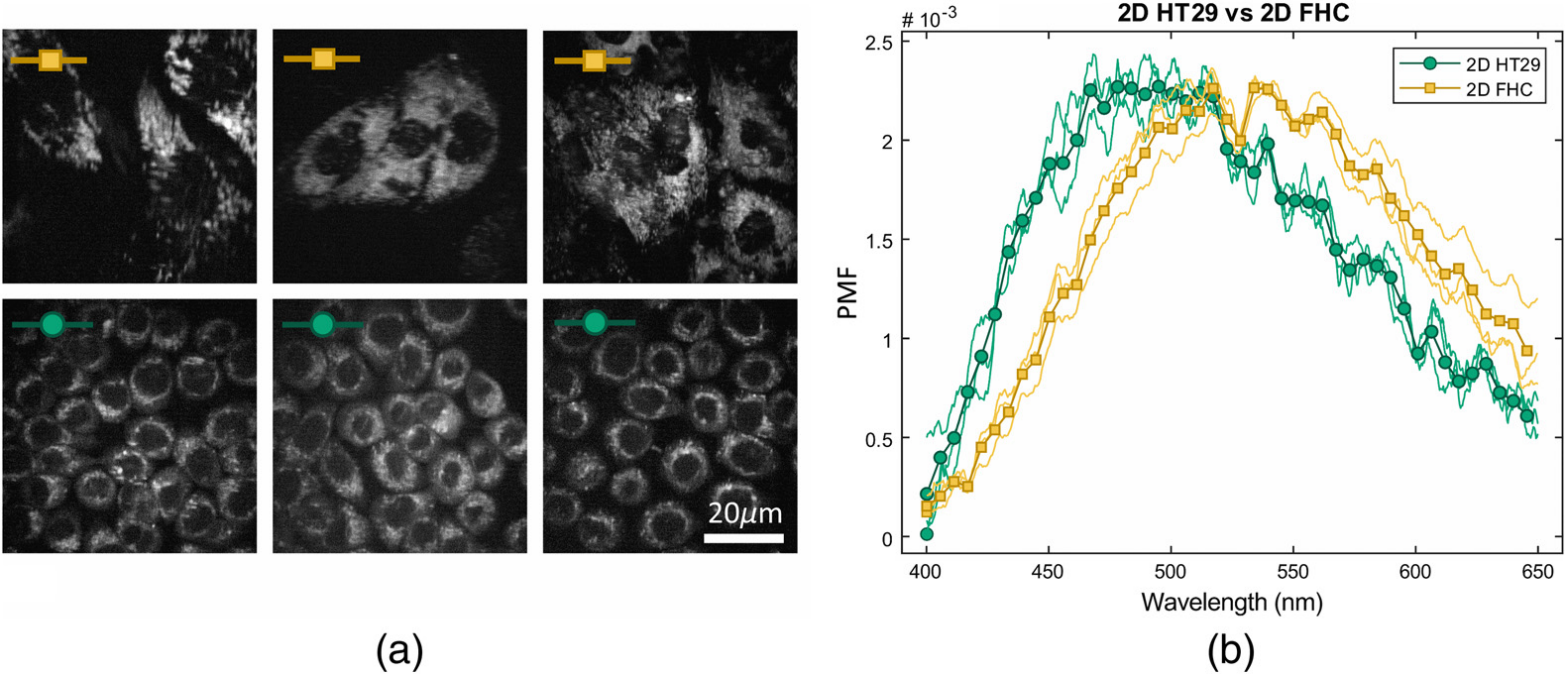
(a) TPEFM images of cancerous (HT29) and normal (FHC) cells in 2D cultures (PMT detection and entire visible spectrum). (b) Corresponding TPEF spectra summed over the full respective images, normalized to the area underneath the curve for better visual comparison.

The obtained TPEF spectra from 2D cell cultures are shown in Fig. 4. TPEFM images of cancerous (HT29) and normal (FHC) cells in 2D cultures (PMT detection, entire visible spectrum) are shown in Fig. 4(a). Figure 4(b) shows the corresponding TPEF spectra summed over the full respective images, normalized to the area underneath the curve for better visual comparison. Figure 4(a) shows the cancerous (HT29) or normal (FHC) cells that are normalized using the PMF for a better visual comparison and plotted in Fig. 4(b) along with the mean spectra. Autofluorescence spectra for cancerous cells are blueshifted when compared to their normal counterparts, which is in agreement with findings in cell cultures^25^ and clinical studies.^26^ To ensure the applicability of the presented method, variations within a sample group need to be smaller than the difference between the sample groups. The shift between FHC and HT29 spectra has been observed despite small changes in culturing parameters for 2D cultures and spheroids as shown in Figs. S5(d) and S5(b) in the Supplementary Material, respectively. A relative variability is defined as described in the Supplemental Material and calculated measurements on 2D cultures are shown in Fig. 4 and measurements on two additional samples are included in Fig. S5(d) in the Supplementary Material. Briefly, the center of gravity varies around 12% for FHC cell cultures and 10% for HT29 cell cultures, compared to the general shift between the cell types. For the spheroid cancer models, the measured spectra follow a similar spectral shift, as shown in Fig. 6(c). Individual measurements are shown in Fig. S5(c) in the Supplementary Material and their relative variabilities calculate to 29% for HT29 and 3% for FHC cells.

### 3.2 NAD(P)H and FAD Autofluorescence

**Fig. 5:**
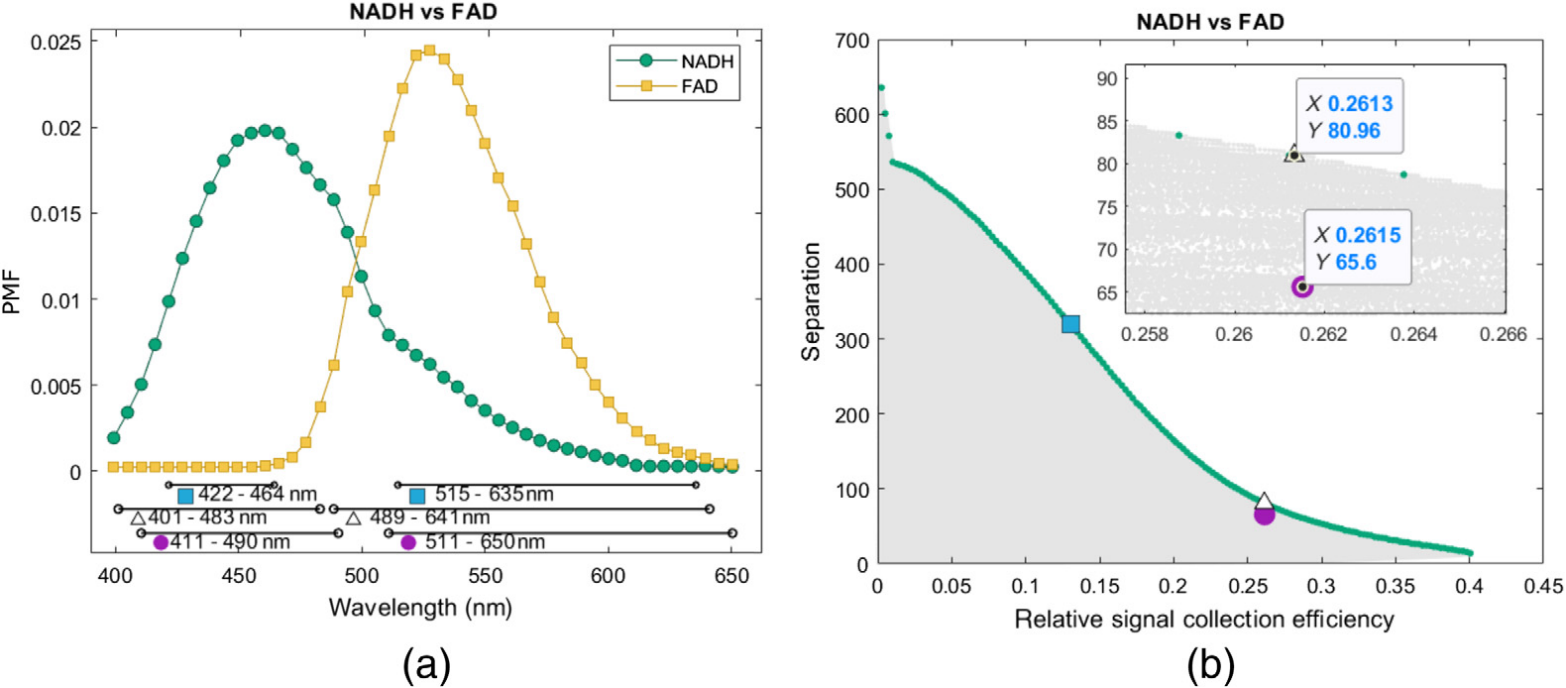
Simulations of spectral bands on data from literature.^17^ (a) Spectra of NAD(P)H and FAD are normalized using the PMF and shown as used for the simulation. Spectral bands of bandpass filters (410 to 490 nm and 510 to 650 nm, circle) and improved spectral bands (401 to 483 nm and 489 to 641 nm, triangle) are marked. Additionally, spectral bands with a high separation and reduced relative signal collection efficiency are marked (square). (b) Simulated combinations of spectral bands are plotted with their separation (scaling the contrast of a system) and their relative signal collection efficiency (scaling the SNR of a system). The combinations of spectral bands shown in (a) are marked and favorable combinations yielding a maximal achievable separation are highlighted.

The algorithm presented above is first verified using TPEF emission spectra of NAD(P)H and FAD data as an example of a possible biomarker from the literature,^17^ as shown in Fig. 5(a). The gray area in Fig. 5(b) represents the different spectral band combinations. For every relative signal collection efficiency, the respective maximal achievable separation lies on the green line. The combination of a 410- to 490-nm and 510- to 650-nm spectral band as used in Ref. 17, marked with a circle, yields a relative signal collection efficiency of 0.26 and a separation of about 65.6. A filter setup resulting in the same relative signal collection efficiency (within 0.1% tolerance) and 23% increased separation is determined and marked with a circle: the corresponding spectral bands are 401 to 483 nm and 489 to 641 nm. The spectral bands of both setups are marked in Fig. 5(a). Due to the relatively small overlap of spectra in Fig. 5(a) (as compared to real autofluorescence spectra), a much stronger separation could potentially be reached when choosing the narrow spectral bands 422 to 464 nm and 515 to 635 nm outside of the overlapping parts of the spectra. A fourfold increase in separation of 320 as opposed to 81 is calculated in this case for an SNR reduced by a factor of 2.

### 3.3 Cell Model Autofluorescence

**Fig. 6:**
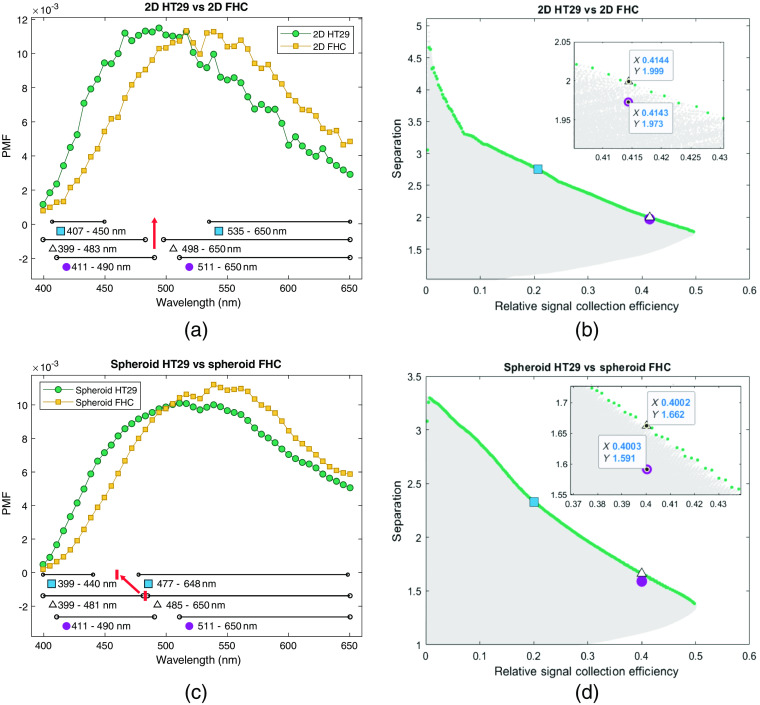
TPEF spectra from different cancer models are investigated and spectral bands with optimized separation are visualized. Autofluorescence spectra of cancerous (HT29) and noncancerous (FHC) cell cultures grown in (a) 2D and (c) spheroid cancer models are shown normalized using the PMF as used for the simulation. Spectral bands of bandpass filters (410 to 490 nm and 510 to 650 nm, circle) and improved spectral bands (triangle) are marked for the investigation of cancer models. Simulated combinations of spectral bands are plotted for (b) 2D and (d) spheroid cancer models with their separation (scaling the contrast of a system) and their relative signal collection efficiency (scaling the SNR of a system). The combinations of spectral bands shown in (a) and (c) are marked and favorable combinations of spectral bands yielding a maximal achievable separation between the measured spectra are highlighted.

Measured autofluorescence spectra of live 2D cell cultures and spheroids, shown with their average spectrum in Figs. 6(a) and 6(c), respectively, are used in the following as inputs for the algorithm to determine optimal detection bands. Evaluating the simulated combinations of spectral bands on 2D cultures in Fig. 6(b), the 410- to 490-nm and 510- to 650-nm bands suggested in Ref. 17 already yield a separation of 1.97, very close to the best possible value of 2 with optimized spectral bands at the same relative signal collection efficiency of 0.41 given by said bands. The same is true for the 3D cultures. The separation of the spheroid cell models can neither be improved by more than 0.07, from 1.59 to 1.66, when keeping the relative signal collection efficiency the same at 0.4, as shown by the inset in Fig. 6(d). It can be seen that, for both cell models and for measurements of NAD(P)H and FAD in solution (above), the determined optimal spectral bands at the same relative signal collection efficiency are shifted (band 1) or expanded (band 2) toward the blue when compared to the 410- to 490-nm to 510- to 650-nm bands suggested by the literature. The expansion of band 2 is considerably stronger in the spheroid cell models than in the 2D cell cultures.

In both cancer models, a significantly improved separation can be achieved by compromising in relative signal collection efficiency. Combinations of spectral bands with increased separation and halved relative signal collection efficiency are marked (square) in Fig. 6(b) for 2D cell autofluorescence and Fig. 6(d) for spheroid autofluorescence. In the case of 2D cell cultures, a separation of 2.7 as opposed to 2 can be achieved compared to the literature bands. Correspondingly, a halved relative signal collection efficiency increases the separation of spheroid autofluorescence from 1.7 to 2.3. In 2D cell cultures and spheroids alike, a better separation requires a particularly narrow spectral band in the blue part of the spectrum and a broader band in the red part of the spectrum.

### 3.4 Imaging Application

**Fig. 7:**
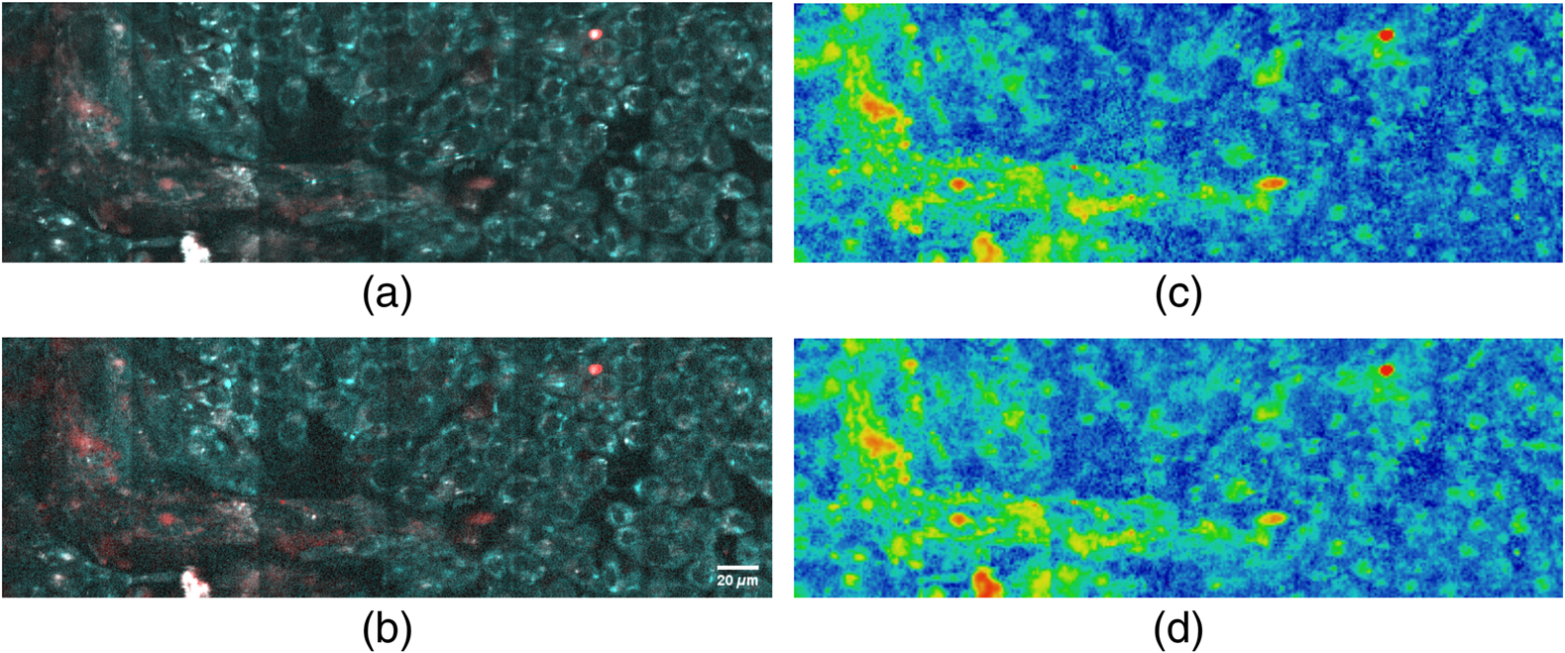
(a) and (b) Color-coded images are calculated from hyperspectral images of a 2D co-culture (HT29 and FHC cells). Images are composed by integrating over (a) initial spectral bands: 410 to 490 nm (cyan), 510 to 650 nm (red) and (b) optimized spectral bands: 407 to 450 nm (cyan), 535 to 650 nm (red). (c) and (d) Ratiometric images are calculated by dividing the red channel by the sum of both channels for (c) the initial and (d) optimized bands. To improve SNR, a 2×2 binning is applied.

To demonstrate the capabilities of the presented method, a mosaic composed of 12 hyperspectral images from an FHC and HT29 2D co-culture is acquired with a resolution of 256 pixel and a pixel dwell time of 20 ms for each frame. Figure 7(a) shows an image composed of two channels generated by integrating over the initial^17^ spectral bands (cyan: 410 to 490 nm and red: 510 to 650 nm). In Fig. 7(b), which is composed by integrating over the optimized spectral bands calculated above (cyan: 407 to 450 nm and red: 535 to 650 nm), an increased noise is visible due to the narrower spectral bands. Despite this, a stronger color contrast is visible, making the FHC cells appear redder and thus more detectable. In both images, both channels are scaled to the same thresholds relative to their histogram (lower limit: mean −2σ, upper limit: mean +5σ, σ: standard deviation). To verify these findings numerically and to provide comparability to the commonly used redox ratio, we divide the red channel by the sum of both channels to create ratiometric images of the initial spectral bands as shown in Fig. 7(c) and the optimized spectral bands are shown in Fig. 7(d). A 2×2 binning is performed to increase the SNR of these images. The SNR of all images is calculated as described by Nylk et al.^27^ in the frequency domain using the 2D fast Fourier transform, by considering features with a spatial frequency higher than four times the theoretical system resolution as noise, while features at lower frequencies were considered to be signal. The SNR is then calculated as the ratio of the integrals over the respective power spectra. While the SNR of the individual image channels decreases by 2.6% (red) and 42.6% (cyan), the calculated SNR of the ratiometric image is increased by 13.1%. Furthermore, a statistical analysis in the form of a Student’s t-test is performed on 12 hyperspectral images of 2D FHC and HT29 monocultures to quantify an improved confidence in differentiating these specimen populations. Aforementioned spectral bands are used to calculate a redox ratio for ease of comparison as detailed in Table S1 and Fig. S6 in the Supplementary Material. Although the difference of the mean redox values between cell cultures only increased by 3.7%, the lower standard deviation of 31.9% (FHC) and 8.3% (HT29) leads to an improved separation when using optimized spectral bands, as shown by combining these values in a T-ratio, which increases by 31.5%.

## 4 Discussion

In this work, we demonstrate a method to define optimal spectral bands for the detection of disease-specific biomarkers. A possible separation of two different TPEF spectra, a measure for the potential diagnostic accuracy, can be calculated as a function of the relative signal collection efficiency, a measure for the expected SNR, also influencing the system’s expected accuracy, such that a realistic trade-off for a diagnostic or application-oriented setup can be chosen. We conclude that the separation of spectra can be optimized for the application, but the resulting bands in our measurements are still comparable among different cell models and NAD(P)H and FAD spectra. This further confirms that these fluorophores are major contributors to the investigated live cell TPEF spectra as reported for other cell lines and tissue types in literature.^2^ It also highlights the advantages of ratiometric measurements for smaller system variations. Since our excitation is around 785 nm as opposed to 800 nm, the two-photon action cross section and thus the NAD(P)H fluorescence is excited by a factor of 3 to 4 more efficiently,^3^^,^^17^ yet the spectral bands in the detection still yield a similar separation. With this being said, the effective signal collection efficiency needs to be considered: If the signal collection can be made more efficient, e.g., by the choice of more sensitive detectors, spectral bands with a higher separation can be chosen to significantly improve the contrast of a ratiometric measurement. In both cases, for the classification of 2D cell cultures and spheroids, the separation is calculated to increase by 35% given an acceptable loss in SNR by a factor of 2 compared to 410- to 490-nm to 510- to 650-nm spectral bands. When applied to a 2D co-culture, the improvement of separation shows as an increase of SNR by 13.1% in a ratiometric image with a decrease of SNR by 42.6% in the more strongly affected individual channel. While the difference in the absolute numbers may be attributed to the method of creating the images and calculating the SNR, the relative increase in contrast for a given compromise in SNR is as expected. A statistical analysis of images obtained from 2D monocultures likewise shows an improvement in separation as an increase of the T-ratio in a t-test by 31.5%.

Interestingly, the overall higher achievable separation for 2D cell cultures compared to spheroids makes them sound like an attractive cancer model as their biomarker for disease diagnostics seems expressed more distinct. However, it should be considered that changes in autofluorescence spectra can be triggered by environmental influences, which are not targeted. Since 2D cell cultures express more cell-to-surface signaling, they may be more susceptible to changes in their environment, such as the cell medium, exposure to room temperature, oxygen, or ambient light, which may all trigger a spectrally changed fluorescence. This also indicates that, for clinically relevant systems, a lower possible separation should be expected, emphasizing the need for the best possible filters allowing the highest diagnostic accuracy.

While investigating pure NAD(P)H and FAD spectra, a strong separation could be theoretically reached. Although in practice the value of separation is strongly influenced by the low intensity of FAD fluorescence below 470 nm (which in this case is a nearest neighbor extrapolation from digitalized data), this still represents a realistic measurement environment and illustrates that in some cases it may be beneficial to reject overlapping spectral components to increase the contrast. This holds especially true in scenarios where SNR is not a limiting factor or can be retrieved by higher excitation powers, as this may be the case for low-bleaching samples outside of a clinical context such as the detection of toxins in food safety.^28^

Counterintuitively, in the particular case of measurements on NAD(P)H and FAD in solution, the achieved separation with the filters proposed in the literature is further away from the optimum than for the cell models. A possible explanation is that in complex tissue environments other fluorophores may significantly influence the autofluorescence spectrum and external influences may alter the individual TPEF spectra.^14^ As shown in Fig. S4 in the Supplementary Material, these changes are dominated by autofluorescence and the effect of scattering and absorption can be neglected in the biological models used for demonstration. Sample variations due to these effects should be considered in an application, where absorption may have an impact (such as single-photon excited fluorescence of vascularized tissue and skin) or where imaging is performed over a great range of depths in scattering tissue.^20^ Despite using the same cell lines for 2D and 3D cell models, the fluorescence spectrum is influenced by the microenvironment of the cells. On the optimal spectral bands defined for spheroid cancer models, a tendency toward the blue part of the spectrum can be seen. With increasing separation, the center between optimal bands (marked with an arrow in Fig. 6) is blueshifted. If the system is not limited due to noise, smaller bands can be evaluated to increase the separation and thus the classification or imaging contrast in a diagnostic context. At the same time, this points toward the importance of the 400- to 450-nm regime of the autofluorescence spectra. It has been shown that cancerous tissue exhibits a blueshifted spectrum compared to normal tissue due to a strong contribution in this spectral band.^29^ While without the support of fluorescence lifetime measurements, further interpretation of this shift is beyond the scope of this study, similar changes have previously been attributed to an altered ratio in bound and free NAD(P)H.^20^ By evaluating this part of the spectrum potentially different biomarkers are probed than the redox ratio, such as hypoxia, which has been linked to the relative contribution of protein-bound NAD(P)H to the autofluorescence spectrum.^30^ Despite the fact that spheroids are created in an effort to express hypoxia, and due to a denser tissue and increased metabolism a stronger hypoxia is expected in the HT29 spheroids, we do not observe a blueshift that would be associated with a hypoxia-induced reduction in protein-bound NAD(P)H. This might be due to the fact that imaging the depth of 10 to 20  μm allows for sufficient diffusion. The potential ability to measure protein-bound and free NAD(P)H without the need for fluorescence lifetime imaging would provide valuable information for cancer diagnosis at an early stage^31^ and can be implemented with reduced complexity and costs in a clinical context. Further studies using the presented method together with fluorescence lifetime imaging could reveal disease-specific spectral bands and establish simple intensity measurements to improve early-stage cancer diagnosis. While a separation into two channels is assumed in this study for simplicity, the algorithm could be extended to accommodate further channels for multiparametric analysis.

## 5 Conclusion

We showed that the presented method has the ability to improve the contrast of a given label-free optical imaging system. We suggest that application-oriented research is conducted to investigate detection setups for a broad range of illumination sources and specific diseases or problems. Interdisciplinary collaborations are necessary to provide access to spectra for specific applications and thus design much needed application-driven advanced imaging systems.^18^

To determine optimal filter sets for a given diagnostic or research system using our algorithm, one should first select samples that represent two different states, which are to be classified, and then a hyperspectral imaging setup has to be used once to acquire the spectra. The spectra can be analyzed by the algorithm to determine the trade-off between separation to signal collection efficiency. This knowledge would finally allow the determination of the optimal filter set for the application.
