# Supplementary material for: Selecting optimal spectral bands for improved detection of autofluorescent biomarkers in multiphoton microscopy
Source: J Biomed Opt. 2020 Jul 7;25(7):071206. doi: 10.1117/1.JBO.25.7.071206 (PMC7338838; doi:10.1117/1.JBO.25.7.071206)
Supplement: Supplementary file 1 [file JBO_025_071206_SD001.pdf]

Supplemental Material

Hyperspectral multiphoton microscopy system

The system is a custom-built Multiphoton Microscope, as shown in Figure S1. A femtosecond laser (Femtosource Compact Pro, Femtolasers GmbH, Austria) with a center frequency at 785 nm, a pulse length of <15 fs and a 74 MHz repetition rate is used for illumination. After passing a dispersion pre-compensation unit (Mosaic Pro V, Femtolasers GmbH, Austria), the beam is sent through a spatial filter (KT130, Thorlabs, USA), with a 15.3 mm focusing lens (C260TMD-B), a 10 µm pinhole and a 50 mm collimation lens (AL2550H-B), thus enlarging the beam. A galvanometer scanner (GVS012, Thorlabs, USA) redirects the laser beam on the sample with the relay lenses (AL2550H-B, AC254-150-B) enlarging the beam further to fill the back aperture of the objective. The beam passes a long pass filter (736/LP, Semrock, USA), is reflected by a dichroic mirror (HC720SP, AHF, Germany) and focused by a water dipping objective (XLUMPLFLN 20XW, Olympus). Emitted light is collected through the same objective, filtered by the dichroic mirror and a short-pass filter (720/SP, Semrock, USA), before being focused into a fiber with a 600 µm core. The signals can then be detected by a PMT (H7422, Hamamatsu, Japan) for quick imaging or a spectrometer (QE Pro, OceanOptics, USA) for hyperspectral imaging. The setup is controlled by a custom LabVIEW program. For hyperspectral imaging, an average power of 12 mW on the samples was used with a pixel dwell time of 8 ms.

Spectral Processing

Spectra of entire hyperspectral TPEF images (64x64 pixels) are averaged for a better signal to noise and smoothened over a range of 10 nm, matching the spectral resolution of the spectrometer given the input slit with (fiber core size) of 600 µm.

All spectra are background subtracted and compensated for a temperature offset using Spectragryph. This is done by averaging the signal in the 705 – 750 nm region and subtracting it as a baseline from the spectrum.

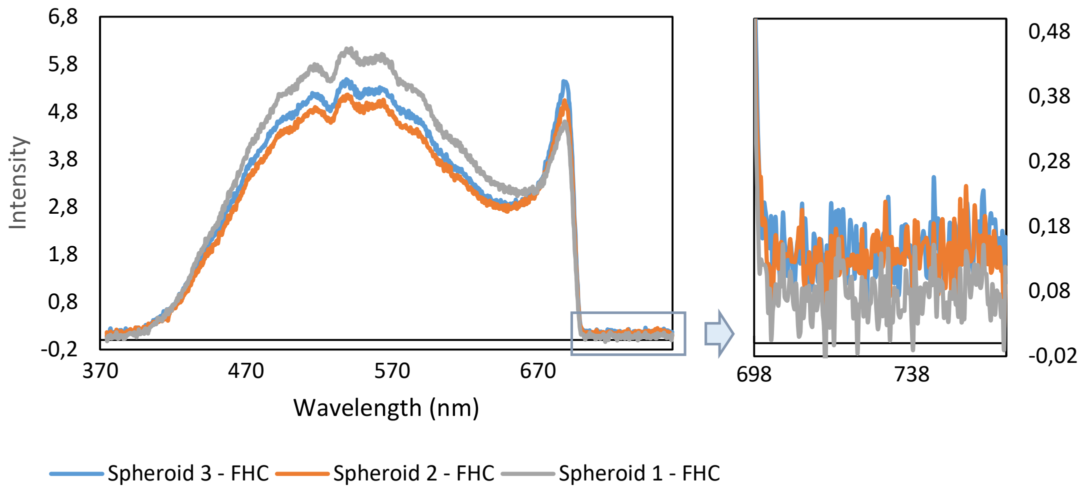

Figure S1: Spectral processing: Due to a slight temperature shift, an offset is introduced. This is corrected using a baseline correction (mean of 705 – 750 nm set to be 0)

Spectral range

The lower boundary of our spectral detection is given by our system response function. After recording autofluorescence spectra of HT29 spheroids with increasing power (extracted from 64x64px images as explained above), the resulting spectra are divided by the strongest spectrum. A response can be seen from 400 – 700 nm. Our excitation at 785 nm limits TPEF slightly lower than obtained by this experiment, further limiting factors might be transmissions of some of the optics.

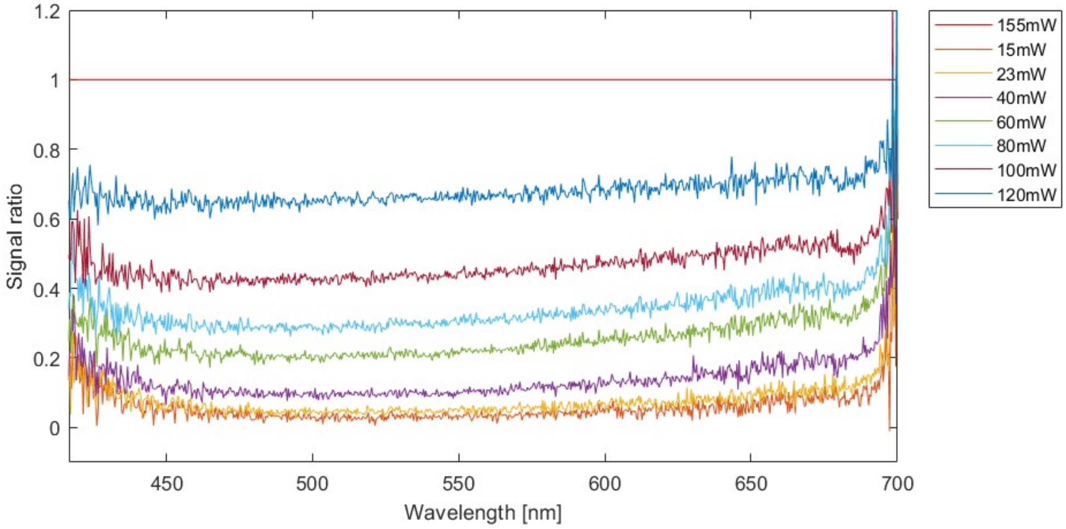

Figure S2: The system response determines the lower boundary of spectrum. The power in the sample plane is reduced to 10% of the given value

Excitation light

The raw spectra show part of the laser excitation in the detection. When comparing acquisitions conducted with and without excitation filter, it can be seen, that the excitation is suppressed by a factor of more than 100 above 650 nm, whereas below no strong laser influence from the laser excitation can be seen. It can be expected, that in this part of the spectrum the laser emission is effectively suppressed. An upper boundary of 650 nm is selected.

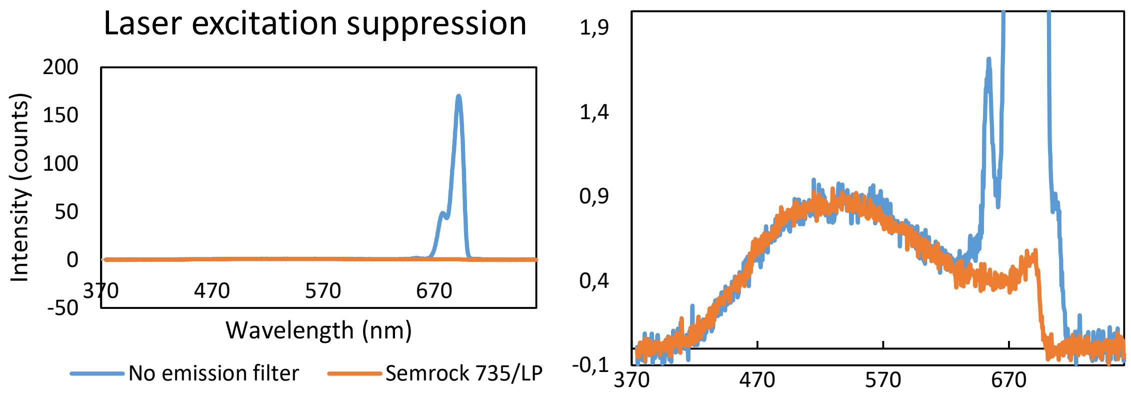

Figure S3: The laser excitation spectrum propagates through the excitation filter at wavelengths above 650 nm. It can be seen that the suppression is effective (factor 200) and no significant influence of laser excitation can be expected below 650 nm

Spectral change due to transmission through tissue

To ensure the sample-dependent influence of absorption and scattering on fluorescent light at the given imaging depth is negligible in the cancer models used throughout this study, light transmission has been investigated. Transmission spectra of entire HT29 and FHC spheroids have been recorded by placing them in focus between two microscope objectives (Illumination: LMPlan 50X IR, Collection: XLUMPLFLN 20XW, Olympus). The sample was illuminated with a halogen lamp (Fiber-Lite PL-800, Dolan Jenner) and transmitted light was detected by a spectrometer (QE Pro, OceanOptics, USA) after passing the aforementioned detection setup including a dichroic mirror (HC720SP, AHF, Germany), short-pass filter (720/SP, Semrock, USA), and fiber with a 600 µm core. For comparison against the illumination source, intensity matched measurements with decreased integration time have been performed, in which the sample was translated out of the illumination path. The spectra were background compensated, and the transmission spectra were multiplied by the ratios of integration times to reproduce a comparable intensity. From these two measurements, the wavelength dependent attenuation coefficient (assuming the diffusion approximation to be applicable) was calculated by solving Equation S1 for $\mu_t(\lambda)$, taking into account the diameter of the spheroid, which is different for the two cell lines (FHC: approx. 270 µm; HT29: approx. 500 µm). To estimate the spectral changes after a transmission through 20 µm of the tissue, the wavelength dependent transmission $T(d,\lambda)$ is then calculated, given this $\mu_t(\lambda)$ and with *d* = 20 µm, from Equation S1. As can be seen in Figure S4, the expected change in spectral shape (intensity) is a maximum of 2.6% (HT29 spheroids, FHC 0.8%), which is negligible in the case of these spheroids, as was confirmed by multiplying the obtained spectra by these transmission curves before feeding them into the presented algorithm, but yielding only an increased separation of 2.7% using obtained bands (constant SNR). Even for a larger imaging depth of 50 µm, the change in spectral shape would still be below 6.6 % (HT29 spheroids, FHC 2%), and we therefore suggest not to compensate the spectra before applying our algorithm, as this is not necessary, but partly also because measuring the wavelength dependent attenuation coefficient, even as crudely as presented here, is not straight-forward in many samples.

$$T(d,\lambda) = \frac{I_{trans}(\lambda)}{I_{ill}(\lambda)} = e^{-\mu_t(\lambda)*d} \qquad \text{(S1)}$$

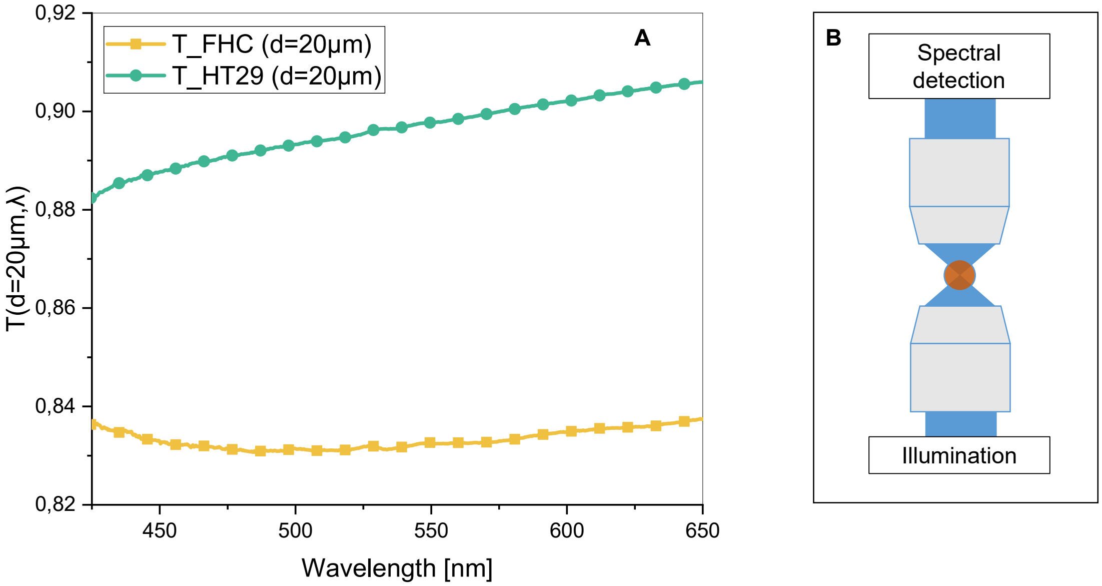

Figure S4: A) Transmission curve for HT29 and FHC spheroids, which could be multiplied by a fluorescence spectrum to simulate a transmission through a tissue at a depth of 20 µm to account for absorption and scattering. B) Setup: Transmission spectra of HT29 and FHC spheroids along with reference spectra (without spheroids) have been recorded by placing the samples in focus between two microscope objectives. Transmission curves are then derived from these measurements.

Reproducibility of autofluorescence spectra

To assess the variability of the fluorescence spectra, a range of parameters was tested to give an estimate about the robustness of the presented method in the described application. Spectra were acquired from multiple samples, using varying excitation power and culturing duration. Figure S5 (a) shows little dependence on the excitation power with some potential bleaching in higher wavelength regions. Although Figure S5 (b) shows only small changes in fluorescence spectra depending on the cultivation method, it can be seen, that the autofluorescence spectra of HT29 spheroids is slightly different. This may be due to variations in the imaging location or variation between sample batches. However, the general shift remains visible. In all cases, a homogenous layer of cells is selected for imaging to avoid influences from debris, single cells and the necrotic core. Figure S5 (c) shows the autofluorescence spectra of spheroids used for demonstrating the method in the main manuscript. To give an estimate on the variability within the data, the center of mass was calculated for all spectra. The difference of a single measurement to the mean spectrum of the corresponding sample type is calculated and divided by the difference of the two mean spectra to calculate a relative variability. The average relative variability in HT29 spectra is 29% compared to 3% in FHC spectra. In Figure S5 (d), the spectra of 2D cultures used in the main manuscript (Petri dishes PD1&PD3, cultured 9 days high oxygen, 3 days low oxygen) are plotted along with another set of measurements on 2D cultures (Petri dishes PD2&PD4, cultured 7 days high oxygen, 4 days low oxygen). Three FOVs per cell line are imaged on each petri dish. The average relative variability of all six spectra, calculated as above, is 12% for FHC spectra and 10% for HT29 spectra.

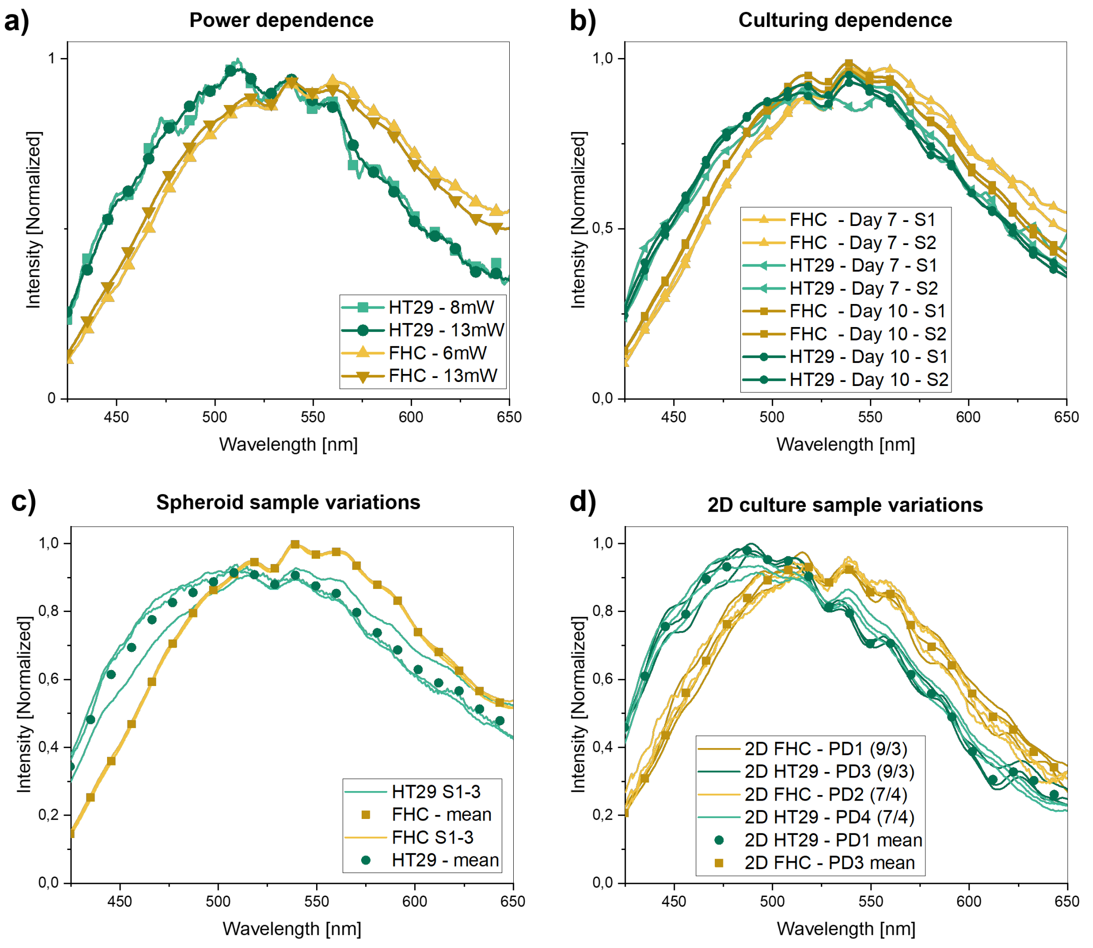

Figure S5: Reproducibility of autofluorescence spectra: a) Autofluorescence spectra of an HT29 and an FHC spheroid at reduced and full illumination intensity on the same FOV for each sample. Samples were grown 7 days at normal (20%) oxygen and 3 days at low (5%) oxygen conditions. b) Spectra were obtained from spheroids grown for 7 days in high oxygen (Day 7) and Spheroids grown for 7 days in high and for 3 days in low oxygen (Day 10). c) Three spheroids per sample type are imaged and the mean spectra are used in the main manuscript. Spheroids were grown for 9 days in low oxygen and 3 days in high oxygen. One FOV is imaged per spheroid. d) Spectra of three FOV of two 2D cultures per sample type: Petri dishes PD1&PD3 (spectra used in the main manuscript, cultured 9 days high oxygen, 3 days low oxygen) Petri dishes PD2&PD4 (cultured 7 days high oxygen, 4 days low oxygen) with three FOVs on one petri dish per sample type.

Statistical analysis of an increased population separation on the example of 2D cultures

For a numerical verification of an increased separation between two specimen populations with our optimized bands, the hyperspectral images of 2D cell cultures, two petri dishes each for FHC (PD1, PD2) and HT29 (PD3, PD4), with their average spectra shown in S5 d), are investigated. In this example, image processing is performed to allow for a comparison to the commonly used redox ratio[8]. After background subtraction and baseline correction as described above, the cytoplasm is selected using an intensity threshold. Subsequently a redox ratio for two sets of spectral bands are calculated as shown in Table S1: In the case of *redox F1*, the sum of intensities of all segmented pixels in the 510 – 650 nm band is divided through the sum of intensities of all segmented pixels in the 410 – 490 nm and 510 – 650 nm bands. Likewise, the 407 – 450 nm and 535 – 650 nm bands are used to calculate *redox F2*. The differences in mean population values $\overline{Redox_{FHC}}$ and $\overline{Redox_{HT29}}$ along with their standard variations are compared as shown in Figure S6. While the difference of the sample mean values increases only by 3.7%, implying that

the populations can be differentiated only very slightly better, the standard deviation is reduced by 31.9% (FHC) and 8.3% (HT29), therefore leading to a higher confidence in separating the populations. In an effort to quantify the improved separation, these values are combined in a T-Ratio, calculated as part of a Student's t-test according to Equation S2 with a sample size *n* of 6.

$$T - Ratio = \frac{\overline{Redox_{\boldsymbol{FHC}}} - \overline{Redox_{HT29}}}{\sqrt{\frac{s_{\boldsymbol{FHC}}{}^2}{n} - \frac{s_{\boldsymbol{HT29}}{}^2}{n}}} \qquad (S2)$$

It can be seen, that population separation increases with narrower spectral bands, as the T-Ratio increases by 31.5%. Although absolute numbers should be interpreted with caution due to the low sample number, this leads to a higher certainty in separation, shown by a greatly decreased p-value.

Sample	Type	Redox F1	Redox F2	Sample	Type	Redox F1	Redox F2
PD1 (9/3) FOV1	FHC	0,701	0,864	PD3 (9/3) FOV1	HT29	0,590	0,732
PD1 (9/3) FOV2	FHC	0,740	0,890	PD3 (9/3) FOV2	HT29	0,570	0,717
PD1 (9/3) FOV3	FHC	0,722	0,877	PD3 (9/3) FOV3	HT29	0,574	0,723
PD2 (7/4) FOV1	FHC	0,730	0,886	PD4 (7/4) FOV1	HT29	0,578	0,728
PD2 (7/4) FOV2	FHC	0,749	0,889	PD4 (7/4) FOV2	HT29	0,609	0,755
PD2 (7/4) FOV3	FHC	0,718	0,865	PD4 (7/4) FOV3	HT29	0,592	0,739
**Statistics**	**Type**	**Redox F1**	**Redox F2**		**Type**		
**Mean**	FHC	0,726	0,879	**Mean**	HT29	0,585	0,732
$\boldsymbol{s_{FHC}}$: **Std. dev.**	FHC	0,0172	0,0117	$\boldsymbol{s_{HT29}}$: **Std. dev.**	HT29	0,0147	0,0135

**T-Test** (two-sided, unequal var.)	**Redox F1**	**Redox F2**
$\overline{\boldsymbol{Redox_{FHC}}} - \overline{\boldsymbol{Redox_{HT29}}}$	0,141	0,146
**T – Ratio**	15,26	20,07
**P – Value**	3,84E-08	2,77E-09

Table S1: Redox ratios calculated from hyperspectral images of 2D cell cultures. Redox F1: 510 – 650 nm band divided by the sum of 410 – 490nm and 510 – 650 nm bands. Redox F2: 535 – 650 nm band divided by the sum of 407 – 450 nm and 535 – 650 nm bands. Petri dishes PD1&PD3 were cultured 9 days high oxygen, 3 days low oxygen, whereas petri dishes PD2&PD4 were cultured 7 days high oxygen, 4 days low oxygen. In all cases, three FOVs per petri dish per cell line are evaluated. An increased confidence in separating the populations using spectral bands F2 is a result of a lower standard deviation as shown by a derived T-Ratio.

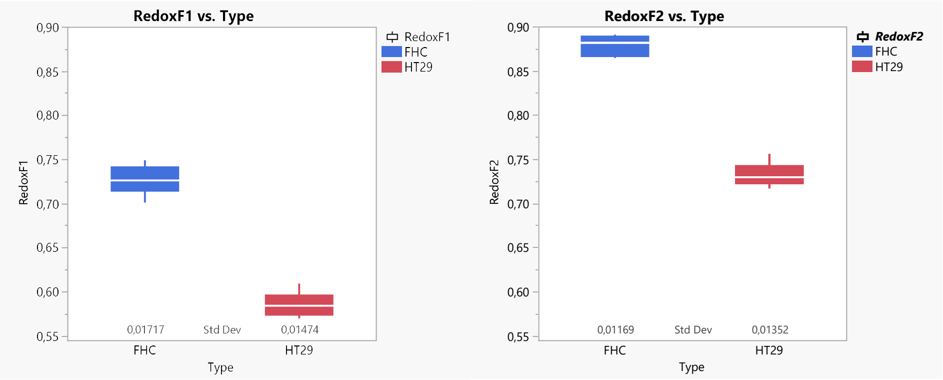

Figure S6: Standard deviation (vertical lines) of redox values visualized along with their mean values (white lines) and quantiles (boxes), derived from measurements on 2D cell cultures described in Table S1. A strong decrease in standard deviation can be observed in FHC cultures using optimized spectral bands F2.
